# HIV-1 Transmission Patterns in Men Who Have Sex with Men: Insights from Genetic Source Attribution Analysis

**DOI:** 10.1089/aid.2018.0236

**Published:** 2019-08-30

**Authors:** Stéphane Le Vu, Oliver Ratmann, Valerie Delpech, Alison E. Brown, O. Noel Gill, Anna Tostevin, David Dunn, Christophe Fraser, Erik M. Volz

**Affiliations:** ^1^ Department of Infectious Disease Epidemiology, National Institute for Health Research Health Protection Research Unit on Modeling Methodology, Imperial College London, London, United Kingdom.; ^2^ Department of Mathematics, Imperial College London, London, United Kingdom.; ^3^ HIV and STI Department of Public Health England's Center for Infectious Disease Surveillance and Control, London, United Kingdom.; ^4^ Institute for Global Health, University College London, London, United Kingdom.; ^5^ Nuffield Department of Medicine, Big Data Institute, Li Ka Shing Center for Health Information and Discovery, University of Oxford, Oxford, United Kingdom.

**Keywords:** age-mixing, HIV epidemiology, phylogenetic, phylodynamics

## Abstract

Near 60% of new HIV infections in the United Kingdom are estimated to occur in men who have sex with men (MSM). Age-disassortative partnerships in MSM have been suggested to spread the HIV epidemics in many Western developed countries and to contribute to ethnic disparities in infection rates. Understanding these mixing patterns in transmission can help to determine which groups are at a greater risk and guide public health interventions. We analyzed combined epidemiological data and viral sequences from MSM diagnosed with HIV at the national level. We applied a phylodynamic source attribution model to infer patterns of transmission between groups of patients. From pair probabilities of transmission between 14,603 MSM patients, we found that potential transmitters of HIV subtype B were on average 8 months older than recipients. We also found a moderate overall assortativity of transmission by ethnic group and a stronger assortativity by region. Our findings suggest that there is only a modest net flow of transmissions from older to young MSM in subtype B epidemics and that young MSM, both for Black or White groups, are more likely to be infected by one another than expected in a sexual network with random mixing.

## Introduction

Men who have sex with men (MSM) account for 40% of new HIV diagnoses in Europe.^1^ In the United Kingdom (UK), nearly 60% of new infections are estimated to occur in MSM, although there is a recent sign of decline in diagnoses particularly recorded in London.^2^ It has been estimated that the largest contribution to transmission in the UK is attributable to young HIV-positive MSM.^3^ More generally, since the early work from Morris *et al.*,^4^ young MSM having sex with older partners have been suggested to increase the risk of infection^5,6^ and to represent a significant driver of the epidemic in North America.^7^ This disassortative age mixing pattern is also considered in interaction with mixing by ethnicity.^8,9^ Among MSM, black men appear to be more affected by HIV in both the UK and US contexts and age mixing patterns have been evaluated to illuminate this ethnic disparity in prevalence.^10–12^ In addition to the question of transmission patterns by age and ethnicity, it is unclear whether the geographic variation in diagnosis rate for MSM is solely reflecting the demographic distribution of groups at greater risk in the country, or can also be explained by a varying extent of transmission between persons of different regions.^13^ Assessing the primary sources of infection in these different demographic groups could prove helpful to design more effective intervention strategies.

Several studies have used phylogenetics to infer transmission patterns based on coclustering of persons from different demographic or risk groups. For instance, occurrences of clustering observed between older and younger MSM is suggestive of a flow of transmission from old to young, as prevalence tends to increase with age.^14,15^

However, there are several limitations to the interpretation of genetic clustering in terms of transmission. Clustering of genetically similar viruses is influenced by time since infection when patients are sampled, which is confounded by patients' age as well as CD4 and clinical stage of infection. Also the extent of clustering is dependent on the fraction of infected persons sampled, which makes direct inference of transmission patterns difficult using genetic clustering.^16–18^ Particularly, the direction of putative transmission events cannot be resolved by pairwise genetic distance alone, and it is not possible to estimate flows of transmission between age groups based on clustering observations.

In this study, we applied a phylogenetic source attribution (SA) method that infers the probability of potential transmission (infector probability) between pairs of patients among ∼15,000 MSM diagnosed in the UK with available genetic sequences.^19^ SA methods based on consensus pol-sequence data cannot be used to infer transmission pairs with high confidence, but can provide useful insights when studied in aggregate over thousands of putative transmission pairs. In general, direction of transmission cannot be inferred from consensus HIV sequence data, but in combination with clinical stage of infection at the time of sequencing, directionality can be inferred probabilistically in some cases, as when for example a patient with chronic infection is linked to a patient with early infection.

By combining phylogenetic analysis with stage of infection data and independent estimates of incidence and prevalence in the population, we are able to quantify potentially imbalanced transmission patterns between different risk groups. To this end, we used sequencing data routinely collected for drug resistance testing, patient-level data informative of the time since infection to account for biased sampling, and population estimates of background prevalence and incidence to account for potentially unsampled individuals that could be the sources of infection. In estimating transmission pair probabilities, our objective was to reveal patterns of transmission in MSM according to age, ethnicity, and geography. In particular, we searched for evidence of source-sink relationships in transmission patterns between age groups and examined the hypothesis that there is a net flow of transmissions from old to young MSM overall or by ethnicity.

## Materials and Methods

### Data

We used partial HIV-1 pol sequences collected in the UK HIV Drug Resistance Database^20^ linked with characteristics of patients newly diagnosed with HIV from the UK Collaborative HIV Cohort study database and the national HIV/AIDS Reporting System database,^21^ as of end of August 2016. Among MSM diagnosed with HIV after 1997 in the UK, 58% had at least one sequence. The data were fully anonymized.

We analyzed adult patients reported as MSM; infected by HIV-1 subtype A1, B, C, or CRF-02AG (the four most represented subtypes); and having a nucleotide sequence while treatment naive. The first sequence per patient with length >950 nucleotides was included. CD4 count values closest to and within a maximum of 1 year of the date of sequence sampling were used to define five stages of infection, comprising early HIV infection (stage 1) and four stages of declining CD4 with thresholds at 500,350 and 200 cells/mm^3^.^22^ In our sample, 81% of patients had a CD4 count. A positive result from the avidity-based recent infection testing algorithm (RITA) led to classifying a patient as at stage 1. Results of RITA at diagnosis were available as of 2009, and from this year were informed for 46% of patients.

Age of patients was categorized in quartiles of age at the date of resistance testing. Difference in age between patients was calculated relative to year of birth. Ethnicity categories were grouped in seven classes: White; Black Caribbean; Black African; Other or unspecified black; Indian, Pakistani, or Bangladeshi (South Asian); Other Asian or Oriental, Other, and mixed. Regions of diagnosis were categorized in five classes: London; South of England; Midlands and East of England; North of England; Northern Ireland, Scotland, and Wales. In analyses of assortativity, unknown category was treated as missing data.

### Sequence processing

Partial HIV-1 pol sequences from the UK were sampled from 1997 to July 2015 with a majority obtained after 2009. Subtypes were determined with REGA version 3.^23^ To infer importation of viral lineages, a BLAST search^24^ was performed for each UK sequence to identify the global sequence from the Los Alamos HIV sequence database (LANL)^25^ with highest similarity. We retained 1,780 unique matching global sequences, as more than one UK sequence may have the same BLAST match. Four reference alignments^26^ per each subtype were also added to UK sequences to serve as outgroup for rooting the phylogenetic trees. All alignments were obtained with MAFFT version 7.^27^ Drug resistance mutation sites were stripped from the alignments.^28^

### Phylogenetic analysis

Phylogenetic trees were constructed with ExaML by maximum likelihood-based inference with a gamma distribution model for rate heterogeneity among sites.^29^ One hundred bootstrap replicates of each tree were computed to account for phylogenetic uncertainty.

We calculated root-to-tip distance and regressed distance by time from MRCA to sample. By iterations of Grubb's algorithm,^30^ we identified on overall 0.3% sequences as outliers in terms of divergence time and evolutionary rate. We applied least-square dating algorithm^31^ on rooted trees and sampling times to estimate the substitution rate and dates of ancestral nodes.

We analyzed separately the four main subtypes to account for different evolutionary rates. Fitch algorithm was used to reconstruct ancestral host status (UK vs. global) and determine distinct clades of virus transmitted in the UK.^32^ The dated subtype B phylogeny comprised 18,484 taxa and for computational reasons was split into subtrees (clades) for further analyses. The tree splitting step consisted in iteratively testing thresholds of forward times (above the root) to slice^33^ the large tree into clades with maximum size of 1,000 taxa (viruses from UK patients). Thus for each of 100 bootstrap trees for subtype B, resulting clades were different.

### Probabilistic source attribution

We applied a phylogenetic SA method that uses a population genetic model to derive probabilities that a given individual (donor) is the source of infection for another individual (recipient) in the sample. These probabilities, termed *infector probabilities*, account for the epidemiological and sampling processes by incorporating into their calculation the time-scaled phylogeny, patient data on stage of infection, and population-level data on occurrence of infection.^19^ The method was evaluated in a previous simulation study.^18^

For population-level epidemic statistics, we used updated incidence estimates of CD4-based back-calculation method for MSM population and prevalence estimates of Bayesian multiparameter synthesis of surveillance data, as reported by Public Health England in 2017.^13^ To account for uncertainty in those input parameters, we randomly drew five pair values of incidence and prevalence per bootstrap replicates (2,000 in total) from normal distributions inferred from the credible intervals of those estimates. Incidence and prevalence were assumed to be proportional across subtypes.

The SA method uses a continuous-time Markov chain model to reconstruct the likely state of a lineage at the time of transmission given the CD4 stage of infection at time of sampling. The definition of stages of infection and progression rates were based on Cori *et al.*,^22^ as described in our previous analysis.^18^ In case of missing CD4 count and missing RITA results at sampling, individuals were assigned a stage with probability relative to the average duration of respective stages. The method assumes that each infected patient corresponds to a single lineage of virus, ignoring multiple infections, and that internal nodes in the phylogeny correspond to a transmission event between hosts. To limit calculations to non-negligible pairing, only coalescent events within a limit of 20 years before sequence sampling were incorporated to compute infector probabilities.

### Statistical procedures

Infector probabilities ${W_{ij}}$ for each donor/recipient pair were averaged over all bootstrap replicates. To compare the mean age of donors and recipients we used a two-tailed paired weighted *t*-test on years of birth, with pair-level infector probabilities as weights.

To characterize transmission patterns by patients' covariates, we first computed a symmetric mixing matrix *M* as the normalized sum of infector probabilities representing aggregated number of transmissions between category *k* $\left( {k = 1 , \ldots , m} \right)$ of recipients and category *l* $\left( {l = 1 , \ldots , m} \right)$ of donors defined by age, ethnicity, and region of diagnosis ($\mathop \sum \nolimits_k^m \mathop \sum \nolimits_l^m {M_{kl}} = 1$). We then calculated three types of output matrices: (1) ${R_{kl}} = {M_{kl}} / \mathop \sum \nolimits_{z = 1}^m {M_{kz}}$, representing the conditional probability for a recipient in category *k* of being infected by a donor in category *l*; (2) ${D_{kl}} = {M_{kl}} / \mathop \sum \nolimits_{z = 1}^m {M_{zl}}$, representing the conditional probability for a donor in category *l* of having transmitted to a recipient in category *k*; and (3) $A = \left( {M - E} \right) / E$, the assortativity matrix representing excessive transmission between categories of donors and recipients relative to random allocation. The matrix *E* has elements ${E_{kl}} = \mathop \sum \;^k {M_{kl}} \otimes \mathop \sum \;^l {M_{kl}} / \mathop \sum \;^k \mathop \sum \;^l {M_{kl}}$, and represents the expected values in the absence of preferential mixing.^34^ Matrix *E* allows the calculation of Newman's assortativity coefficient $r = \left( {Tr \left( M \right) - Tr \left( E \right) } \right) / \left( {1 - Tr \left( E \right) } \right)$. The coefficient ranges from −1 to 1, where $r = 0$ when there is no assortative mixing, $r = 1$ when there is perfect assortativity (every link connects individuals of the same type), and some negative value $- 1 \le r < 0$ for a perfectly disassortative network. In all matrix-type figures, we represent transmission going from donors in columns to recipient in rows.

### Code availability

The code used in this article is available as a R package: https://github.com/slevu/garel

## Results

### Characteristics of the study population

The demographic and geographic composition of the 19,847 HIV-1 partial pol sequences from treatment-naive patients diagnosed in the UK is described in Table 1. Most gay and bisexual men diagnosed in the UK were infected with subtype B (93%). Therefore, the patterns of transmission inferred from reconstructed phylogeny of subtype B sequences are largely dominating that of all MSM patients. Patients infected with non-B subtype were on average sampled later (median year of 2008 for subtype B, 2009 for subtypes A1 and C, and 2011 for CRF02AG) and were on average younger (median age of 35 for subtype B, 34 for subtypes A and C, and 32 for CRF02AG).

**Table 1.** Characteristics of the Study Population

		*A*		*B*		*C*		*CRF02AG*		*All*	
*Subtype*		n	*(%)*	n	*(%)*	n	*(%)*	n	*(%)*	n	*(%)*
Year of sampling	[-Inf, 2002]	17	4	1,867	10	18	3	5	2	1,907	10
	[2002, 2007]	128	29	6,497	35	188	31	47	15	6,860	35
	[2007, 2012]	186	42	7,652	41	303	50	171	53	8,312	42
	[2012, Inf]	107	24	2,468	13	94	16	99	31	2,768	14
Age group	[16, 30]	151	34	4,946	27	196	33	145	45	5,438	27
	[30, 37]	95	22	5,160	28	152	25	62	19	5,469	28
	[37, 44]	89	20	4,303	23	129	21	57	18	4,578	23
	[44, 85]	103	24	4,075	22	126	21	58	18	4,362	22
Ethnicity	White	377	86	15,664	85	417	69	177	55	16,635	84
	Black Caribbean	4	1	408	2	14	2	23	7	449	2
	Black African	19	4	199	1	67	11	52	16	337	2
	Black other/unspecified	3	1	186	1	20	3	12	4	221	1
	Indian/Pakistani/Bangladeshi	7	2	265	1	25	4	6	2	303	2
	Other Asian/Oriental	11	3	549	3	19	3	20	6	599	3
	Other/Mixed	12	3	625	3	24	4	20	6	681	3
	Other	2	0	285	2	12	2	3	1	302	2
	Not known	3	1	303	2	5	1	9	3	320	2
Region of birth	UK	247	56	9,489	51	249	41	136	42	10,121	51
	SS Africa	19	4	379	2	81	13	33	10	512	3
	Other	73	17	3,207	17	107	18	91	28	3,478	18
	Not known	99	23	5,409	29	166	28	62	19	5,736	29
Region of diagnosis	London	174	40	9,417	51	269	45	229	71	10,089	51
	ML_E_England	23	5	1,892	10	59	10	31	10	2,005	10
	N_England	117	27	2,309	12	70	12	18	6	2,514	13
	S_England	56	13	2,559	14	113	19	30	9	2,758	14
	NI_S_W	31	7	784	4	32	5	6	2	853	4
	Not_known	37	8	1,523	8	60	10	8	2	1,628	8
	All	438	100	18,484	100	603	100	322	100	19,847	100

In terms of ethnicity, the majority (84%) of patients were white persons. Patients infected with C or CRF02AG were more commonly of non-white ethnicity: Black African for 11% and 16% and from other non-white ethnicity for 19% and 26%, respectively.

In terms of geography, half of subtype B and 71% of subtype CRF02AG sequences were sampled in Greater London. Apart from London, subtype A was especially prevalent in North of England (27%).

### Infector probabilities

Across 100 bootstrap tree replicates for each subtype, we computed infector probabilities for on average 554,514 potential transmission pairs involving 14,603 patients (Table 2). The remaining 5,244 individuals from the initial sample, besides 250 outliers in tree reconstruction, could not be connected by a probability of transmission due to their isolation in distinct clades or the time limit imposed to coalescent event. Although the distribution of infector probabilities is varying across bootstrap replicates, almost all estimates are very small (Supplementary Fig. S1). This confers a very low confidence in any particular pair and interpretations in terms of transmission are only applicable at a group level. Given the *n* by *n* matrix of probabilities that a patient *i* transmitted to a patient *j*, the sum $\mathop \sum\;^i \;{W_{ij}}$ represents the probability that the infector of *j* is in the sample. This quantity, denoted “in-degree”, indicates that on average 36.6% (95% CI [35.2–38.0]) of potential donors are included in our sampled population (Table 2). Our estimates of in-degrees were moderately influenced by the variation in inputs of background incidence and prevalence, with lower incidence (or higher prevalence) increasing average in-degrees as the probability of an unsampled intermediary transmitter is decreased (Supplementary Fig. S2).

**Table 2.** Phylogenetic Reconstruction and Source Attribution Results by Subtype

*Subtype*	*A*	*B*	*C*	*CRF02AG*	*All*
Number of global sequences	199	831	612	138	1,780
Number of sequence outliers	6	163	7	74	250
Median TMRCA (year)	1951	1966	1961	1975	NA
Number of UK patients either donors or recipients	337	13,665	346	255	14,603
Number of infector probabilities estimated between potential transmission pairs	19,818	521,811	6,350	6,535	554,514
Mean in-degree (%)	39.4	36.7	32.6	28.9	36.6

Results are averaged over 100 bootstrap replicates. Global sequences are unique sequences from Los Alamos HIV sequence database matching UK sequences from a BLAST search. Outliers are UK sequences identified as outliers in root-to-tip regression. Mean in-degree represents the probability that the donor of a given recipient is included in the sample.

### Age difference between donors and recipients

**FIG. 1.**
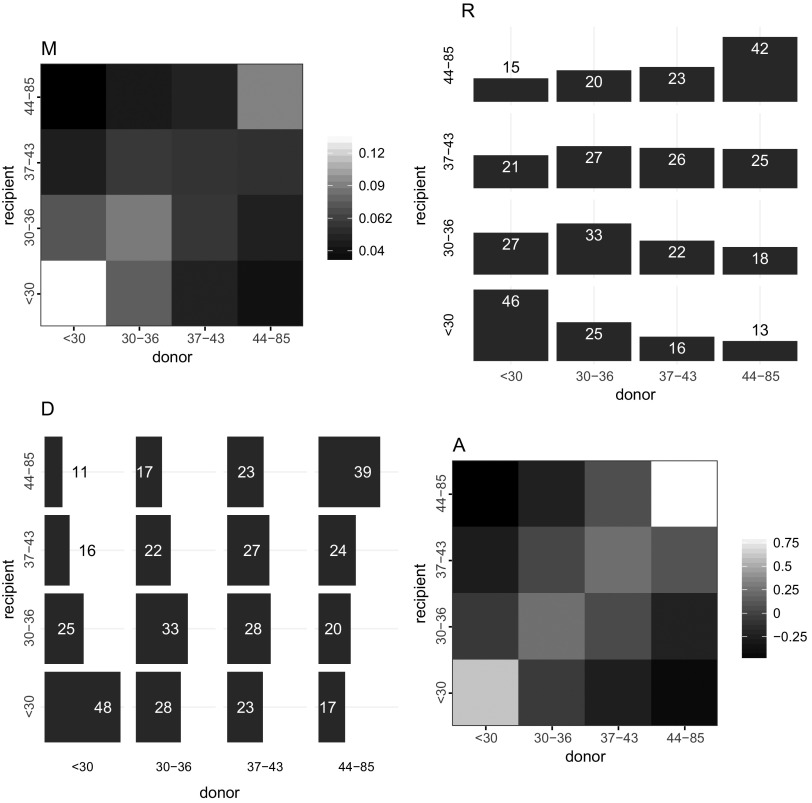
Patterns of transmission of HIV subtype B by age in quartiles. The four graphics depict transmission from donor categories in *column* to recipient categories in *row* (from *x*-axis to y-axis). Axes labels represent ranges of quartiles of age. (**M**) Each cell represents the proportion of overall transmissions from one category to another, with higher proportion in *lighter shade*, that is, the highest amount (14%) of transmissions involved donors and recipients both less than 30 years of age. (**R**) Each *row* represents the probability distribution for a given age category of recipients of having been infected by donors by age, that is, 25% of recipients less than 30 years of age were infected by donor 30–36 years of age. (**D**) Each *column* represents the probability distribution for a given age category of donors of having transmitted to recipients by age, that is, 28% of donors 37–43 years of age, infected recipients 30–36 years of age. (**A**) The assortativity matrix indicates that, relative to random mixing more transmissions occurred within the same age category, particularly for the oldest and youngest. Assortativity coefficient *r* = 0.16.

Table 3 shows the mean difference in age between donors and recipients, weighted by infector probabilities. A significant difference is only detectable for subtype B, donors being on average less than 8 months older than recipients. For subtype B, most transmission pairs in our sample involved individuals less than 30 years of age (Fig. 1M). The largest proportion (46%) of infection acquired by young individuals was attributable to individuals in the same age category (Fig. 1R). And a strong assortativity in transmission mixing is seen in this youngest age category, indicating that young MSM are preferentially infected by young MSM. This preferential mixing is also seen among individuals over 44 years. The overall assortativity coefficient was moderate with $r = 0.16$. Similar transmission patterns between age groups were observed for subtypes A and C (Supplementary Table S1). However, transmission of subtype CRF02AG was characterized by a strong assortativity mostly in the oldest age category but more intergenerational mixing between other categories (Supplementary Fig. S3A). Despite the lack of significant difference in average age of donors relative to recipient shown previously for subtype CRF02AG, the most probable infector for individuals from intermediate age quartiles (30–36 and 37–43) was younger (less than 30) (Supplementary Fig. S3R).

**Table 3.** Difference in Year Between Age of Donor and Age of Recipient

*Subtype*	*A*	*B*	*C*	*CRF02AG*
Age difference^*^	0.13 [−0.80; 0.60]	0.63 [0.53; 0.73]	0.20 [−0.39; 0.71]	0.33 [−0.34; 1.03]
Birth year of donor	1,973.8 [1,973.2; 1,974.5]	1,972.1 [1,971.9; 1,972.2]	1,974.5 [1,973.8; 1,975.1]	1,977.0 [1,975.9; 1,978.6]
Birth year of recipient	1,974.0 [1,972.4; 1,974.7]	1,972.7 [1,972.6; 1,972.8]	1,974.7 [1,974.2; 1,975.0]	1,977.3 [1,976.4; 1,978.8]
Positive difference^**^ (*n*)	30	100	28	47
Negative difference^**^ (*n*)	5	0	3	3
Age at sampling of donor	35.3 [34.7; 36.2]	36.3 [36.2; 36.4]	34.8 [34.2; 35.5]	33.4 [31.9; 34.6]
Age at sampling of recipient	35.4 [34.8; 37.2]	35.9 [35.8; 35.9]	34.8 [34.4; 35.3]	33.2 [31.8; 34.0]

Results are averaged across 100 bootstrap replicates and intervals are 2.5 and 97.5 percentiles.

^*^ Age difference is calculated relative to year of birth.

^**^ Number of *p*-values <.05 for two-tailed weighted *t*-test of the age difference, either positive (donor older than recipient) or negative (donor younger than recipient).

### Transmission by ethnicity

**FIG. 2.**
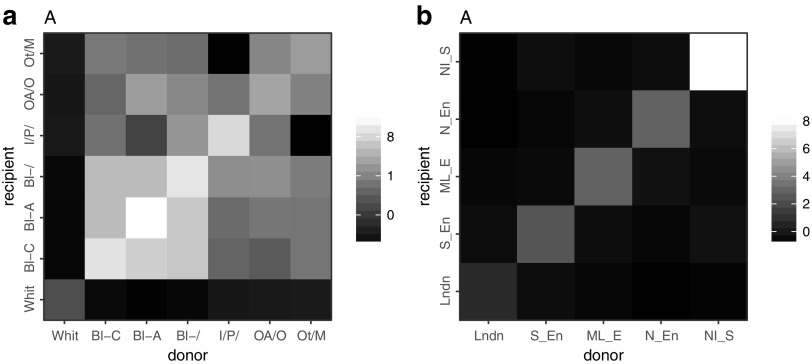
Assortativity in transmission of HIV-1 subtype B by ethnicity and region of diagnosis. *Lighter shades* represent higher assortativity. **(a)** Ethnicities: White; Black Caribbean (Bl-C); Black African (Bl-A); Other or unspecified black (Bl-); Indian, Pakistani, or Bangladeshi (I/P/); Other Asian or Oriental (OA/O), Other and mixed (Ot/M). Assortativity coefficient *r* = 0.17. **(b)** Regions: London, South of England; Midlands and East of England; North of England; Northern Ireland, Scotland, and Wales. Assortativity coefficient *r* = 0.56.

The vast majority (85%) of MSM infected with subtype B viruses were of white ethnicity. We estimated that 82% of all transmissions in our sample occurred between white individuals, and that recipients of all ethnicities had a majority of white donors. The probability of having been infected by a white individual was 92% for whites, 77% for Indian/Pakistani or Bengladeshi, 75% for other Asians, 55% for Black Africans and 54% for Black Caribbean. Conversely, a majority of transmission originating from donors of any ethnic group was estimated to affect white recipients. Figure 2a shows the level of assortativity in transmission of subtype B viruses between ethnic groups. Interethnic transmission (cumulated pair probabilities outside the diagonal) represented 17% on overall and 58% when excluding the white category. Overall assortativity was moderate ($r = 0.17$), but a preferential mixing was especially observed within and between all black ethnic groups and within the South Asian group.

**FIG. 3.**
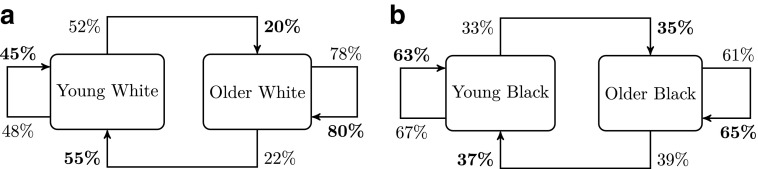
Patterns of transmission of HIV-1 subtype B between young MSM (less than 30) and older MSM by ethnicity: **(a)** White, **(b)** Black (including Black Africans, Black Carribean, and other and unspecified Black). Percentages represent conditional probability of transmitting to recipient type per donor type (*normal font*) and of acquiring infection from donor type per recipient type (*bold font*).

We estimated the probability of transmission of subtype B viruses between young (<30) and older MSM (30+) either from white or black ethnicity (Fig. 3). The relative excess of transmission within age categories observed previously is observed for both white and black ethnicities, and overall assortativity by age was similar ($r = 0.25$ for white and 0.28 for black). However, for a given older MSM, the probability of transmitting to a young MSM was higher in black (39%) than in the white ethnic group (22%).

### Transmission by geographical region

**FIG. 4.**
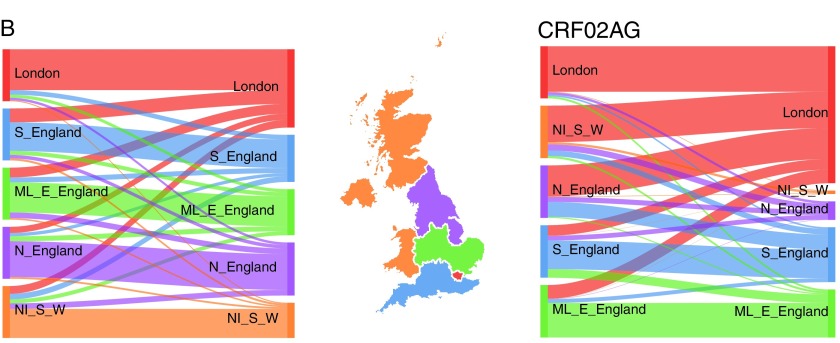
Patterns of transmission of HIV-1 subtype B (*left*) and CRF02AG (*right*), by geography. Each flow diagram, obtained from D matrix described in Methods section, has connections proportional to the probability of transmission from a donor given his region (*left side*) to recipients from respective regions (*right side*). The map is colored by groups of region of diagnosis: London, South of England (S_England); Midlands and East of England (ML_E_England); North of England (N_England); Northern Ireland, Scotland, and Wales (NI_S_W). Color images are available online.

Analyses of transmission by region show the largest level of assortativity, indicating an overall strong spatial structure of the epidemics (Fig. 2b). Assortativity coefficients were 0.56 for subtype B and 0.49 for subtype CRF02AG. For those two subtypes, Figure 4 shows the probability for a donor in a given region to transmit to a recipient of each respective region. For subtype B (left), the majority of transmissions (at least 60%) occur within the same region but donors from every region contributed to infections diagnosed in London (10% for North of England, Northern Ireland, Scotland, and Wales, 20% for the Midlands and East England, and 30% for the South of England). For subtype CRF02AG, there was a higher probability for donors from North of England (60%) or Northern Ireland, Scotland, and Wales (70%) to infect recipients in London than individuals within the same region.

## Discussion

The objective of this study was to describe patterns of HIV transmission between age, ethnicity, and geographical categories in the United Kingdom. We used a phylodynamic inference based on sequences collected among diagnosed MSM, which accounts for incomplete sampling and stage of infection at sampling time. By modeling an epidemic process that is compatible with the evolution of transmitted viruses and epidemiological surveillance data, we characterized past transmission events among nearly 15,000 MSM patients at the national level.

Pair probabilities averaged over phylogenies and aggregated by age groups indicated a modest overall net flow of transmission from older to young MSM. This result is compatible with other studies reporting coclustering of young and older patients^14,15^ as we do not observe pure assortative mixing, with probable transmission occurring in both directions across age groups. But our results indicate that on average, flow from old to young is mostly compensated by the transmission from young to old (Fig. 1). And when the flow is imbalanced, as for transmission of subtype B viruses, the difference is small. We observed an overall preferential mixing in transmission by age with greater assortativity both in the youngest and oldest age groups and more random mixing in intermediate age groups. Understanding age mixing patterns in transmission can help to determine which groups are at a greater risk and potentially guide public health interventions.^35^ Our findings confirm that young MSM infect one another more than expected by random mixing, which supports the idea that prevention benefit could be enhanced by focusing on this small group.^36^ This result also corroborates the observation of recent clusters of young MSM sustaining the epidemic in the Netherlands.^37^

We showed an overall preferential pairing by ethnicity in conjunction with an important mixing between white men and men from other ethnicity. It can be explained by the overwhelming proportion of white men in the population. But in non-white groups, more than a half of transmission was interethnic, revealing that a substantial amount of transmission has occurred between ethnic groups among MSM. A similar pattern for sexual partnership between ethnic groups was reported in Britain.^10^ Although we found a relatively higher assortativity among black MSM in general and a non-negligible mixing between black ethnic groups from different origins (African, Caribbean, and other), HIV transmission appears less assortative among black MSM in the UK than it is in the USA.^38^ We assessed whether intergenerational transmission was different in white and black MSM and found a similar level of age assortativity in both groups. Therefore as others in the US context^9^ we did not find support in our findings to explain a disparity in HIV prevalence by age mixing.^7,8^

Finally, we found a strong geographical structure for the epidemics among MSM, with region of diagnosis as the variable associated with the highest level of assortativity. This implies that interventions in a particular location would take time to diffuse to a wider population. It should be noted that region of diagnosis can be different than the region of residency or of actual transmission, which may lead to an underestimation of the true level of geographical structure.

Several potential limitations of our study relate to the assumptions of the phylogenetic inference and SA method. First, as stated in Methods section, the SA method neglects some effects of within-host evolution, which can cause discordance between phylogenies and transmission trees.^39^ This approximation is reasonable if within-host evolution generates coalescence time considerably shorter than between hosts at the population level. Second, we incorporated crude estimates of incidence and prevalence in the inference of infector probabilities. These were assumed constant over the period and proportional across subtypes. However, variation of these inputs within credible limits had limited impact on average infector probabilities (Supplementary Fig. S2). Third, the direction in transmission was derived from CD4 count and RITA result data that were partially complete.

Nevertheless, our analysis aimed to improve the use of phylogenetic information relative to genetic clustering in two ways. First, by providing a rough measure of transmission probability, which unlike linkage into clusters can indicate a directionality and gives more weight to pairs with higher credibility. Notably, output matrices and patterns between groups would be symmetrical if based on clustering. Second, by correcting for biases stemming from incomplete sampling of the infected host population. Lastly, the SA method was fast to compute and scaled easily to phylogenies based on many thousands of sequences. The approach we take is generalizable to many different settings and has wider applicability to other large pathogen sequence databases.

Future directions for this work include applying the analysis to the heterosexual population, where phylogenetic information could contribute to assess age disparity in mixing across gender.^40,41^ Another direction would be to use methods exploiting next-generation sequencing that account for within-host evolution and enhance resolution in identifying transmission.^39,42^

In conclusion, this study has leveraged available patients data and viral sequences to provide evidence of assortativity in HIV transmission by age, ethnicity, and geography. Understanding these patterns of transmission is important to modeling the impact of intervention strategies.

## Supplementary Material

Supplemental data

Supplemental data

Supplemental data
