# Supplementary material for: HIV-1 Transmission Patterns in Men Who Have Sex with Men: Insights from Genetic Source Attribution Analysis
Source: AIDS Res Hum Retroviruses. 2019 Aug 30;35(9):805–13. doi: 10.1089/aid.2018.0236 (PMC6735327; doi:10.1089/aid.2018.0236)
Supplement: Supplemental data [file Supp_FigureS1.pdf]

Supplementary Data

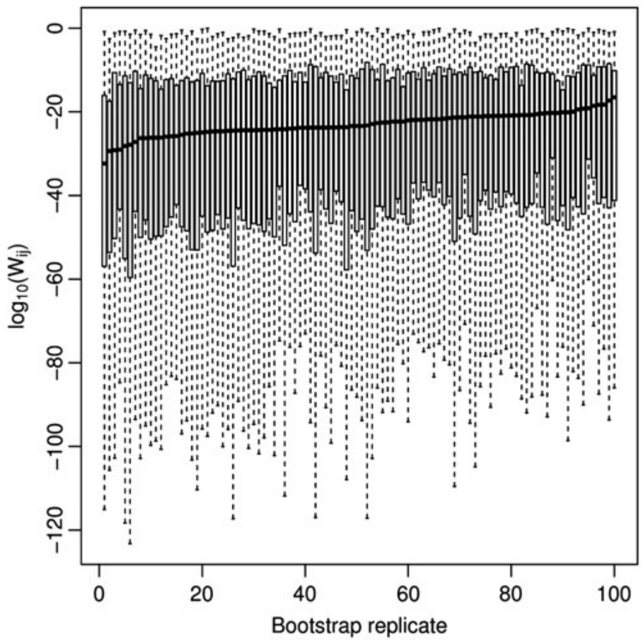

SUPPLEMENTARY FIG. S1. Distribution of infector probabilities across 100 bootstrap replicates. To illustrate the variability of estimates between bootstrap replicates, a random sample of 100 probabilities (in logarithm) per replicate is represented. Results are sorted in increasing order of the median value. Outliers are not shown.
