# Supplementary material for: HIV-1 Transmission Patterns in Men Who Have Sex with Men: Insights from Genetic Source Attribution Analysis
Source: AIDS Res Hum Retroviruses. 2019 Aug 30;35(9):805–13. doi: 10.1089/aid.2018.0236 (PMC6735327; doi:10.1089/aid.2018.0236)
Supplement: Supplemental data [file Supp_FigureS3-TableS1.pdf]

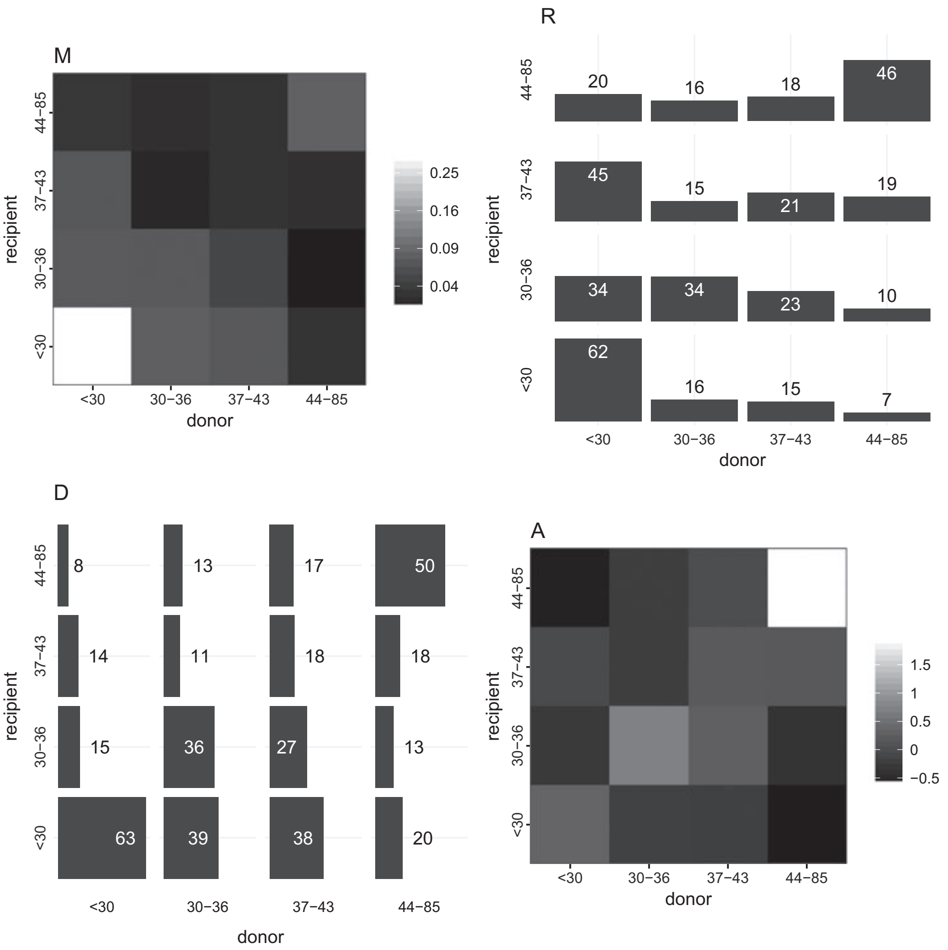

SUPPLEMENTARY FIG. S3. Patterns of transmission of HIV-1 subtype CRF02AG by age. See reading notes from Figure 1 in the main text.

Supplementary Table S1. 95% Confidence Intervals for Proportion of Transmission by Age Quartiles

		M				*R*				*D*			
Subtype	*Age quartile*	*30*	*37*	*44*	*85*	*30*	*37*	*44*	*85*	*30*	*37*	*44*	*85*
A	85	03;08	03;05	06;09	06;09	14;27	12;20	24;34	26;40	09;25	15;23	27;46	31;43
	44	03;04	02;04	04;07	06;09	16;23	11;20	21;34	33;45	08;12	10;18	20;29	28;39
	37	07;09	04;08	02;05	03;04	33;45	19;35	08;25	12;20	19;25	19;35	10;23	11;19
	30	15;23	07;11	04;06	03;05	47;60	19;28	10;16	07;15	44;61	35;48	19;28	13;21
B	85	03;03	04;05	05;05	09;09	14;16	20;21	22;23	41;43	11;12	16;17	22;24	38;40
	44	04;05	06;06	05;06	05;06	20;22	26;28	25;27	24;26	15;16	21;23	26;28	23;24
	37	07;07	09;09	06;06	05;05	26;28	32;34	21;23	17;19	24;25	32;34	27;29	20;21
	30	14;14	07;08	05;05	04;04	45;47	24;26	15;16	12;14	47;49	28;29	22;24	16;18
C	85	01;03	03;05	03;04	05;09	07;16	17;30	17;28	34;51	03;07	10;18	14;22	30;49
	44	05;07	05;07	07;10	03;06	20;27	21;30	28;38	13;22	13;19	19;27	36;47	19;31
	37	08;11	05;09	04;06	03;05	31;43	21;33	16;24	11;20	23;31	21;31	21;32	16;30
	30	17;21	08;13	02;04	01;03	50;61	25;37	06;11	04;09	48;57	32;44	10;19	08;17
CRF02AG	85	02;05	02;04	01;04	05;11	13;30	10;24	11;26	31;58	05;10	09;19	08;23	35;61
	44	05;08	01;04	02;04	01;04	33;53	09;23	16;30	10;25	09;18	07;19	13;26	10;27
	37	05;09	05;09	04;07	01;03	27;41	28;41	17;29	06;15	12;19	28;47	20;37	08;19
	30	25;34	05;10	05;09	02;04	55;69	11;23	10;19	04;09	56;68	29;49	29;46	13;28

M, R and D columns refer to the matrices described in Methods section and represented in Figure 1. Column labels for donors and row labels for recipients represent the upper bound of quartiles of age.
