# Supplementary material for: HIV-1 Transmission Patterns in Men Who Have Sex with Men: Insights from Genetic Source Attribution Analysis
Source: AIDS Res Hum Retroviruses. 2019 Aug 30;35(9):805–13. doi: 10.1089/aid.2018.0236 (PMC6735327; doi:10.1089/aid.2018.0236)
Supplement: Supplemental data [file Supp_FigureS2.pdf]

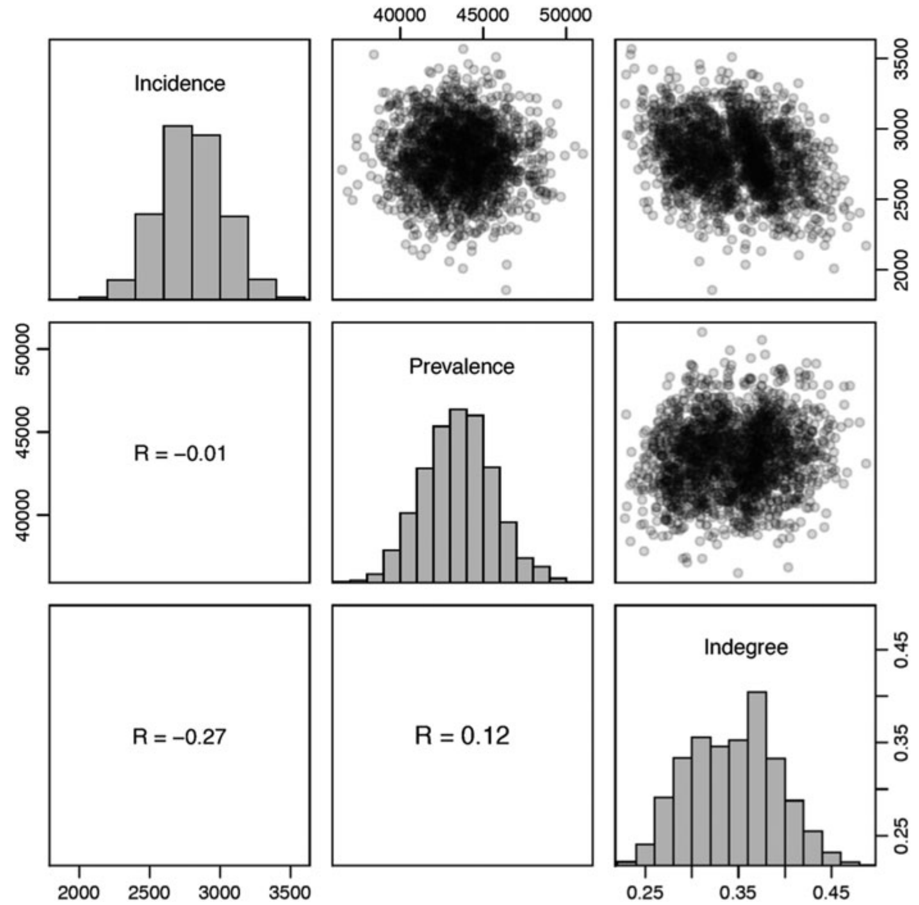

SUPPLEMENTARY FIG. S2. Influence of variation in incidence and prevalence inputs on mean in- degree. Incidence (number of new infections per year) and prevalence (number of persons living with HIV) were drawn independently five times per each of 100 phylogenetic trees for subtype B transmission. Under the source attribution model, the in-degree is the sum of infector probabilities incoming to an individual. It also represents the probability that its infector is in the sample. The mean in-degree tends to increase as prevalence is increased or as incidence is lowered, as it decreases the probability of an unsampled source case. Both variations have a limited impact on the average in-degree estimates as indicated by correlation coefficient R.
